# Comparative Value of the Novel Age-Agnostic DIPSS-R Versus the DIPSS for Prognostication in Myelofibrosis: A Multicenter Evaluation and Reclassification Study

**DOI:** 10.3390/cancers18132159

**Published:** 2026-07-05

**Authors:** Marko Lucijanić, Davor Galušić, Vlatka Periša, Ivan Zekanović, Martina Morić Perić, Hrvoje Holik, Ena Sorić, Rajko Kušec, Danijela Leković, Ivan Krečak

**Affiliations:** 1 Hematology Department, University Hospital Dubrava, 10000 Zagreb, Croatia; 2 Scientific Research and Translational Medicine Department, University Hospital Dubrava, 10000 Zagreb, Croatia; 3 School of Medicine, University of Zagreb, 10000 Zagreb, Croatia; 4 University Hospital Center Split, 21000 Split, Croatia; 5 University Hospital Center Osijek, 31000 Osijek, Croatia; 6 Faculty of Medicine, University of Osijek, 31000 Osijek, Croatia; 7 General Hospital Zadar, 23000 Zadar, Croatia; 8 General Hospital Dr. Josip Benčević, 35000 Slavonski Brod, Croatia; 9 University Clinical Center of Serbia, 11000 Belgrade, Serbia; 10 School of Medicine, University of Belgrade, 11000 Belgrade, Serbia; 11 General Hospital of Šibenik-Knin County, 22000 Šibenik, Croatia; 12 University of Applied Sciences Šibenik, 22000 Šibenik, Croatia; 13 School of Medicine, University of Rijeka, 51000 Rijeka, Croatia

**Keywords:** prognostication, myeloproliferative neoplasms, risk stratification, reclassification, allogeneic stem-cell transplantation

## Abstract

Background/Objectives: The recently proposed revised Dynamic International Prognostic Scoring System (DIPSS-R) excludes age and constitutional symptoms and relies exclusively on disease-related variables. We aimed to externally validate the DIPSS-R and to characterize how it reclassifies patients relative to the DIPSS. Methods: We retrospectively studied 285 patients with primary or secondary myelofibrosis from seven centers. Both scores were calculable in 270 patients (148 deaths). The two systems were compared for discrimination, reclassification, calibration, and robustness across pre-specified subgroups and landmarks. Results: During a median follow-up of 90.3 months, median overall survival (OS) was 66.1 months (5-year OS 52.7%). The two systems showed similar discrimination (C-index 0.691 vs. 0.697; difference −0.006, 95% CI −0.046 to +0.030; *p* = 0.77) with no significant difference across any subgroup, fibrosis grade, driver mutation, age or sex. The DIPSS-R assigned more patients to higher-risk categories (59.3% vs. 46.3%; *p* < 0.001). Reclassification was discordant in 57 patients (21.1%); the DIPSS-R up-stratified 46 patients (17.0%) classified as lower-risk by the DIPSS, and these patients had significantly worse survival than concordantly lower-risk patients (5-year OS 57.7% vs. 81.7%; *p* < 0.001). Among transplant-eligible patients aged ≤65 years, the DIPSS-R up-stratified 26 (22.6%). The reclassification was driven by the DIPSS-R-specific variables (monocytosis, leukocytosis and thrombocytopenia). In multivariable analysis the DIPSS-R remained independently prognostic of the Charlson comorbidity index (CCI), with both retaining independent value (DIPSS-R: HR 1.96 per category, 95% CI 1.58–2.44, *p* < 0.001; CCI: HR 1.18 per point, 95% CI 1.06–1.32, *p* = 0.003). In secondary myelofibrosis the DIPSS-R discriminated comparably to the disease-specific MYSEC-PM (C-index 0.659 vs. 0.698; *p* = 0.44). Conclusions: The DIPSS-R matches the DIPSS in discrimination while identifying additional adverse-risk patients, most relevantly transplant-eligible younger patients, who would otherwise be considered lower-risk by the age-containing DIPSS; in secondary myelofibrosis it performs comparably to the disease-specific MYSEC-PM.

## Simple Summary

Myelofibrosis is a chronic myeloproliferative neoplasm (MPN), developing de novo or as a progression of previous non-fibrotic MPN. Highly variable outcomes and a significant proportion of patients with dismal clinical course, mandate accurate risk estimation to guide type and intensity of therapies. The Dynamic International Prognostic Scoring System (DIPSS), based on both host and disease-related factors, has been used for more than a decade. Age-agnostic revision DIPSS-R was recently proposed. We compared both systems in 285 patients from seven hematology centers. Two systems had similar level of prognostic accuracy across all of investigated subgroups, but the DIPSS-R assigned a substantially larger proportion of patients to higher-risk categories. This phenomenon was especially important among patients younger than 65/70 years of age who may represent a transplant-eligible cohort and in whom earlier and more intensive treatment may improve outcomes.

## 1. Introduction

Myelofibrosis (MF), encompassing primary myelofibrosis (PMF) and secondary myelofibrosis (SMF) arising from polycythemia vera (post-PV) or essential thrombocythemia (post-ET), is a BCR::ABL1-negative myeloproliferative neoplasm characterized by clonal myeloproliferation, bone-marrow fibrosis, ineffective hematopoiesis and a markedly heterogeneous clinical course [1,2]. The disease is driven by largely mutually exclusive mutations in JAK2, CALR or MPL that constitutively activate JAK-STAT signaling [3,4,5,6]. Survival ranges from a few months to more than two decades, and treatment decisions (most critically the timing of allogeneic stem-cell transplantation (alloSCT), the only potentially curative modality) depend heavily on accurate risk estimation [7]. Pharmacologic therapy, principally JAK inhibitors such as ruxolitinib, reduces splenomegaly and symptom burden but is not curative and does not reliably eradicate the malignant clone [8,9].

For more than a decade, the Dynamic International Prognostic Scoring System (DIPSS) has been the most widely applied tool for risk assessment in MF because, unlike the original International Prognostic Scoring System (IPSS) [10], it can be applied at any point during the disease course [11]. The DIPSS allocates points for age > 65 years, leukocytes > 25 × 10^9^/L, circulating blasts ≥ 1%, constitutional symptoms, and hemoglobin < 100 g/L. Although robust, the DIPSS incorporates two elements that are not intrinsic to the disease: chronological age, a host-related factor, and constitutional symptoms, a subjective factor. The strong weight carried by age in particular can dominate the score in older patients and, conversely, leave disease-driven risk under-recognized in younger patients. The DIPSS was subsequently improved by addition of cytogenetic and molecular variables into its framework, resulting in DIPSS-plus [12], MIPSS70 [13], MIPSS70+ v2.0 [14], and the entirely genetics-based GIPSS [15]. However, DIPSS retained its global applicability and wide use due to its simplicity, robustness and non-reliance on expensive and delayed molecular information.

To address aforementioned limitations of host-related and subjective variables, Tefferi et al. recently proposed a revised, age-agnostic clinical model, the DIPSS-R [16]. The DIPSS-R relies exclusively on disease-related variables: two grades of anemia and of leukocytosis, circulating blasts, platelet count, and the absolute monocyte count (AMC), and deliberately omits age and constitutional symptoms. It was developed and internally validated in two independent cohorts, but, as a newly introduced model, it requires external validation in independent, real-world populations. Also, clinical value of the novel score depends on whether and in which direction it reclassifies patients relative to the current standard, and whether those reclassifications are prognostically correct [17].

We therefore conducted a multinational, multicenter study aiming to externally validate the discriminatory performance and calibration of the DIPSS-R relative to the DIPSS across clinically relevant subgroups, and to quantify and characterize how the DIPSS-R reclassifies patients, with particular attention to transplant-eligible patients aged ≤65 years.

## 2. Materials and Methods

### 2.1. Patients and Study Design

We retrospectively evaluated 285 consecutive adult (≥18 years) patients with MF managed at six Croatian hematologic centers (University Hospital Dubrava, University Hospital Center Split, University Hospital Center Osijek, General Hospital Zadar, General Hospital Dr. Josip Benčević Slavonski Brod, and General Hospital Šibenik) and one Serbian center (University Clinical Center of Serbia), diagnosed between June 2004 and February 2026. All patients were Caucasian. Patients had pre-fibrotic or overt PMF, or SMF following PV (post-PV) or ET (post-ET). Diagnoses were reassessed according to the 2022 World Health Organization criteria [2], and bone-marrow fibrosis was graded per the European consensus [18]. The study was approved by the Institutional Review Boards of all participating centers and was conducted in accordance with the Declaration of Helsinki.

### 2.2. Prognostic Scores

Both scores were computed using their original definitions [11,16] at the time of evaluation (which coincided with diagnosis in 262 patients [91.9%]), and overall survival was measured from this same date. A sensitivity analysis restricted to patients scored at diagnosis was performed and yielded essentially identical scores discrimination as in full dataset. Comparative analyses were restricted to the 270 patients in whom both scores were calculable. The DIPSS-R could not be computed in 14, and the DIPSS could not be computed in one additional patient due to unavailable input parameters. These patients did not differ in baseline characteristics from the comparative analysis set, and their exclusion is unlikely to have biased the results. For the DIPSS, one point was assigned for age > 65 years, leukocytes > 25 × 10^9^/L, circulating blasts ≥ 1%, and constitutional symptoms, and two points for hemoglobin < 100 g/L; patients were stratified into low (0 points), intermediate-1 (1 point), intermediate-2 (2–3 points), and high-risk (4–6 points) categories. For the DIPSS-R, one point was assigned for hemoglobin 80–99 g/L (women) or 90–109 g/L (men), leukocytes 11–24.9 × 10^9^/L, circulating blasts ≥ 2%, platelets < 150 × 10^9^/L, and AMC ≥ 1 × 10^9^/L, and two points for transfusion dependence or hemoglobin < 80 g/L (women)/<90 g/L (men) and for leukocytes ≥ 25 × 10^9^/L; patients were stratified into low (0 points), intermediate-1 (1 point), intermediate-2 (2–3 points), and high-risk (4–7 points) categories [16]. Among SMF patients, MYSEC-PM was calculated by original criteria [19].

### 2.3. Statistical Analysis

The Shapiro–Wilk test assessed the normality of numerical variables; because most were non-normally distributed, they are summarized as medians and interquartile ranges (IQR) and compared with the Mann–Whitney U test. Categorical variables are presented as frequencies and percentages and compared with the χ^2^ test; the McNemar test was used to compare paired proportions between the two systems. Overall survival (OS) was estimated by the Kaplan–Meier method and compared with the log-rank test [20,21].

Discrimination was quantified by Harrell’s concordance index (C-index) with 95% confidence intervals (CI) obtained by bootstrap resampling [22]. The difference in C-index between the two correlated models was tested by a paired bootstrap (2000 resamples), and additionally by time-dependent area under the receiver-operating-characteristic curve (AUC) at 12, 36 and 60 months [23]. Reclassification was evaluated by the category-based net reclassification improvement (NRI) and the integrated discrimination improvement (IDI) at 36 and 60 months, with inverse-probability-of-censoring weighting and bootstrap CIs [17,24]. Calibration was assessed by comparing the Kaplan–Meier-observed 3- and 5-year survival across risk categories. Robustness was examined through pre-specified subgroup analyses (MF subtype, fibrosis grade, age, sex, driver mutation), a PMF-restricted sensitivity analysis, and landmark analyses among 12- and 24-month survivors. We additionally estimated, within each subgroup, the hazard ratio for up-stratified versus concordantly lower-risk patients (Cox regression); compared the DIPSS-R with the MYSEC-PM in SMF; and assessed independent prognostic value in multivariable Cox models adjusting for age, comorbidity, MF subtype, sex and driver mutation. Two-sided *p* < 0.05 was considered significant. Analyses were performed in Python 3 (lifelines, scikit-survival, statsmodels) and MedCalc v23.4.5 (MedCalc Software Ltd., Ostend, Belgium).

## 3. Results

### 3.1. Patient Characteristics

A total of 285 patients were analyzed: 90 with pre-fibrotic PMF, 106 with overt PMF, 46 with post-PV SMF, and 43 with post-ET SMF. The median age was 68 years (IQR 60–75) and 110 patients (38.6%) were female; 198 (69.5%) carried the JAK2 V617F mutation, 23 (8.1%) a CALR mutation, 6 (2.1%) an MPL mutation, 38 (13.3%) were triple-negative, and the driver mutation status was undetermined in 20 (7.0%). Baseline characteristics for the overall cohort and stratified by age (≤65 vs. >65 years) are presented in Table 1. As expected, older patients had a higher comorbidity burden [25] (median Charlson index 4 vs. 2; *p* < 0.001), lower hemoglobin and higher absolute monocyte counts, whereas MF subtype, fibrosis grade and JAK2 status were comparable across age groups. During a median follow-up of 90.3 months, 161 deaths occurred; median OS was 66.1 months and the 5-year OS rate was 52.7%. Both scores were calculable in 270 patients (148 deaths), who constituted the comparative analysis set. During the first year of follow-up, the most frequently administered disease-directed therapies were hydroxyurea (58.1%), ruxolitinib (20.4%), anagrelide (6.5%) and interferons (5.4%).

### 3.2. Discrimination and Calibration

**Figure 1:**
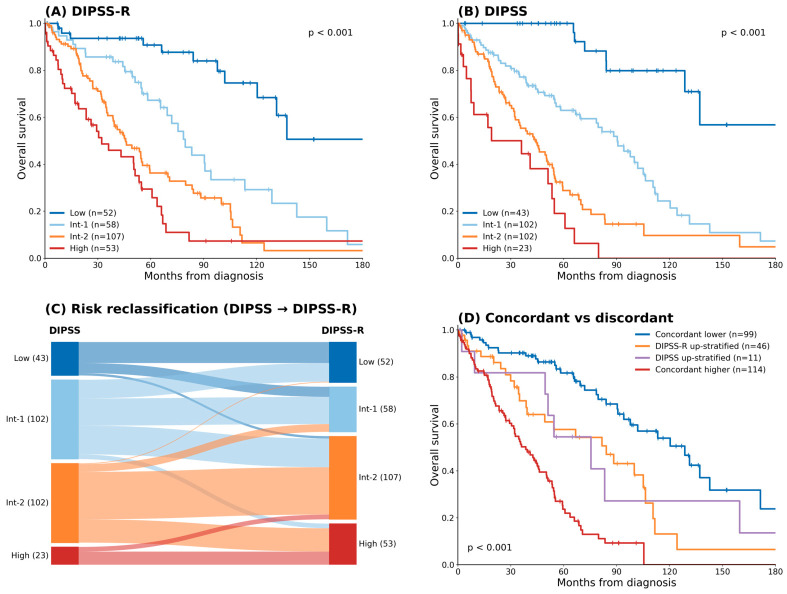
Kaplan–Meier estimates of overall survival and risk reclassification in the overall cohort (n = 270). Overall survival stratified by (**A**) the DIPSS-R and (**B**) the DIPSS. (**C**) Alluvial diagram of risk reclassification from the DIPSS (left) to the DIPSS-R (right); ribbon color denotes the DIPSS-R category. (**D**) Overall survival by concordance/discordance between the two systems; discordantly stratified patients show an intermediate prognosis between concordantly lower- and higher-risk patients (log-rank *p* < 0.001).

**Figure 2:**
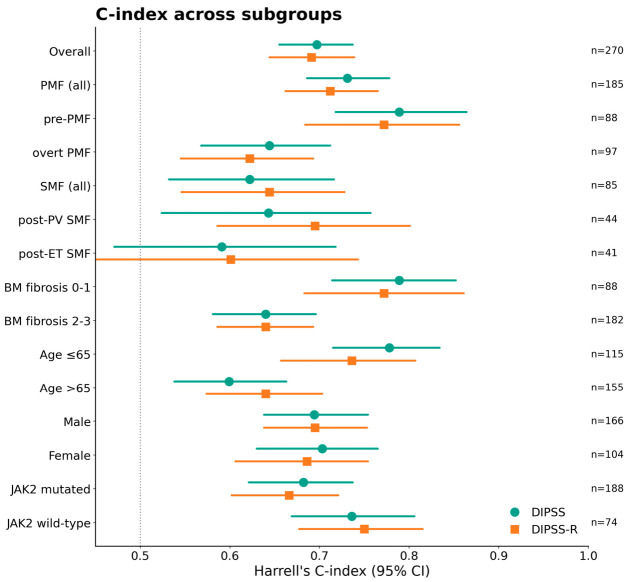
Harrell’s C-index (95% CI) for the DIPSS and the DIPSS-R across subgroups. Overlapping 95% CIs indicate no significant difference in discrimination.

Both systems adequately stratified OS (Figure 1A,B; log-rank *p* < 0.001 for each). Discrimination was moderate-to-good and virtually identical: the C-index was 0.691 (95% CI 0.644–0.739) for the DIPSS-R and 0.697 (95% CI 0.655–0.737) for the DIPSS, with a non-significant difference (DIPSS-R minus DIPSS −0.006, 95% CI −0.046 to +0.030; *p* = 0.77). Time-dependent AUC values were likewise comparable at 12, 36 and 60 months (DIPSS 0.72/0.72/0.79 vs. DIPSS-R 0.71/0.74/0.76; Supplementary Figure S1A), and the IDI did not favor either model (−0.03, 95% CI −0.12 to +0.03). The category-based net reclassification improvement (NRI), comparing the four-category assignments of the DIPSS-R against the DIPSS, was +0.18 (95% CI +0.00 to +0.35) at 36 months and +0.07 (95% CI −0.11 to +0.24) at 60 months. These modestly positive values were driven by the event component (correct upward movement of patients who subsequently died; +0.30 and +0.22 at 36 and 60 months) and partly offset by the non-event component (−0.12 and −0.15), and did not reach statistical significance, consistent with the comparable discrimination and the reclassification analyses described below. Across every pre-specified subgroup (MF subtype, fibrosis grade, age, sex and JAK2 status) the 95% CIs of the two C-indices overlapped, indicating no significant difference in discrimination (Supplementary Table S1, Figure 2). Both models performed best in pre-fibrotic PMF (C-index 0.77–0.79) and least well in overt PMF and post-ET SMF.

Calibration analysis showed that both systems observed survival monotonically across categories (Supplementary Figure S1B). Notably, the two systems differed in the granularity of this separation: the DIPSS defined small but prognostically extreme low- and high-risk groups (observed 5-year OS 100% and 19%, respectively), whereas the DIPSS-R distributed patients more evenly and produced larger, somewhat less extreme low- and high-risk groups (observed 5-year OS 91% and 30%, respectively). This pattern is consistent with the broader high-risk classification produced by the DIPSS-R (see Section 3.3).

### 3.3. Risk Distribution and Reclassification

The distribution of patients across risk categories differed significantly between the two systems (Supplementary Table S2). The DIPSS-R classified a substantially larger proportion of patients into the higher-risk (intermediate-2 plus high) categories than the DIPSS (160/270, 59.3% vs. 125/270, 46.3%; McNemar *p* < 0.001), and nearly doubled the proportion assigned to the high-risk category (19.6% vs. 8.5%). The two systems were concordant in 213 patients (78.9%): 99 (36.7%) concordantly lower-risk and 114 (42.2%) concordantly higher-risk. Reclassification was discordant in 57 patients (21.1%): the DIPSS-R up-stratified 46 patients (17.0%) who were classified as low or intermediate-1 by the DIPSS, whereas the DIPSS up-stratified only 11 patients (4.1%) relative to the DIPSS-R. The directionality and magnitude of these flows are visualized in Figure 1C; the complete cross-classification is provided in Supplementary Table S3.

### 3.4. Clinical Course of Discordantly Stratified Patients

To determine whether reclassification was prognostically correct, we compared survival across the four concordance/discordance groups (Figure 1D; full group characteristics in Table 2). Discordantly stratified patients displayed an intermediate prognosis, lying between concordantly lower- and higher-risk patients. The 46 patients up-stratified by the DIPSS-R (but considered lower-risk by the DIPSS) had significantly worse survival than concordantly lower-risk patients (5-year OS 57.7% vs. 81.7%; median OS 84.1 vs. 128.5 months; log-rank *p* < 0.001), indicating that the DIPSS-R correctly identified adverse-risk patients that the DIPSS missed. Compared with concordantly lower-risk patients, these up-stratified patients were characterized by lower hemoglobin (median 120 vs. 134 g/L; *p* = 0.007), higher leukocytes (16.1 vs. 9.6 × 10^9^/L; *p* < 0.001), higher AMC (0.9 vs. 0.45 × 10^9^/L; *p* < 0.001), lower platelets (387 vs. 528 × 10^9^/L; *p* = 0.03) and more frequent transfusion dependence (10.9% vs. 0%), that is, by the disease-related variables that the DIPSS-R introduces and the DIPSS does not capture.

### 3.5. Reclassification in Transplant-Eligible Patients and Across Subgroups

**Figure 3:**
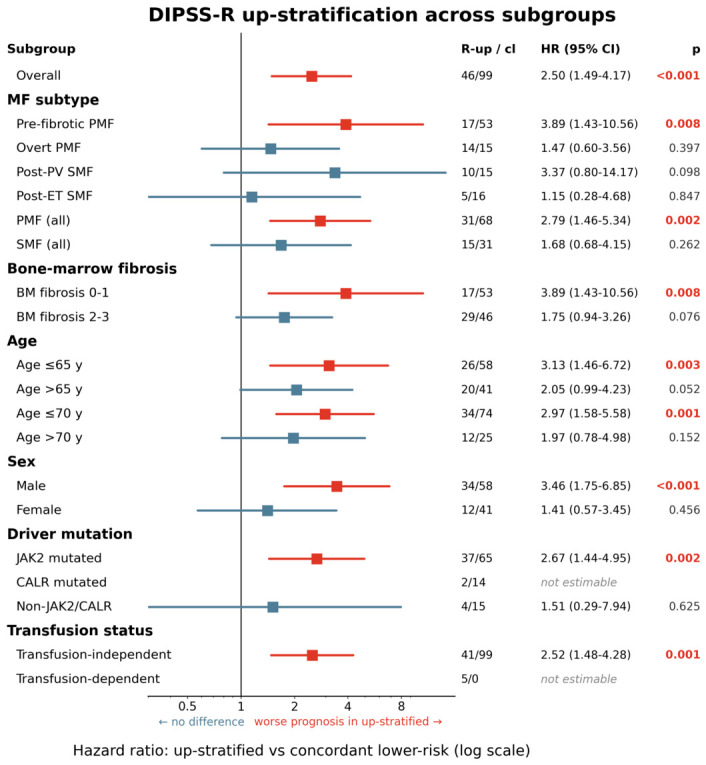
Hazard ratios (95% CI) for death among patients up-stratified by the DIPSS-R (DIPSS-R higher-risk, DIPSS lower-risk) relative to concordantly lower-risk patients, across subgroups. HR > 1 indicates that the DIPSS-R correctly identified worse-prognosis patients; red denotes *p* < 0.05. R-up/cL = number of up-stratified vs. concordant lower-risk patients; not estimable indicates no concordant lower-risk patients or too few events.

Because the age-agnostic design of the DIPSS-R is expected to be most consequential in patients eligible for alloSCT, we focused first on the 115 patients aged ≤65 years, in whom no age-related point can accrue under the DIPSS. In this subgroup the DIPSS-R reclassified 26 patients (22.6%) into the intermediate-2/high-risk categories that the DIPSS had placed in low/intermediate-1, a clinically meaningful proportion of potential transplant candidates whose disease-related risk would otherwise be under-recognized. A comparable effect was observed using a ≤70-year threshold (34 patients, 20.0%). To determine whether this upward reclassification was prognostically correct, we estimated, within each subgroup, the hazard ratio (HR) for death of patients up-stratified by the DIPSS-R relative to concordantly lower-risk patients (Figure 3; Supplementary Table S4). Up-stratification by the DIPSS-R identified significantly worse-prognosis patients among transplant-eligible patients aged ≤65 years (HR 3.13, 95% CI 1.46–6.72) and ≤70 years (HR 2.97, 1.58–5.58). By contrast, the 11 patients up-stratified by the DIPSS rather than by the DIPSS-R were substantially older (median age 76 years) and did not have significantly worse survival than concordantly lower-risk patients (log-rank *p* = 0.85), indicating that the upward classification produced by the DIPSS in older patients largely reflects chronological age rather than disease aggressiveness. Consistent with this, in patients aged >65 years the DIPSS-R retained discrimination (C-index 0.64) whereas the DIPSS performed less well (0.60). Survival curves for the ≤65-year subgroup are shown in Supplementary Figure S2.

Across the remaining subgroups, DIPSS-R up-stratification likewise identified significantly worse-prognosis patients in men (HR 3.46, 1.75–6.85), in JAK2-mutated patients (HR 2.67, 1.44–4.95), in pre-fibrotic PMF (HR 3.89, 1.43–10.56), in PMF overall (HR 2.79, 1.46–5.34) and in transfusion-independent patients (HR 2.52, 1.48–4.28). The effect was not statistically demonstrable in women (HR 1.41, 0.57–3.45), in post-ET SMF (HR 1.15) or in overt PMF (HR 1.47), the latter two subgroups being small and already enriched for higher-risk disease.

### 3.6. Comparison with the MYSEC-PM in Secondary MF

Because the DIPSS-R was not specifically developed for SMF, we compared it with the SMF-specific MYSEC-PM in the 74 SMF patients in whom both scores were calculable (Supplementary Table S5). Both scores significantly stratified overall survival (Supplementary Figure S3A,B). The MYSEC-PM showed numerically higher discrimination (C-index 0.698, 95% CI 0.620–0.773) than the DIPSS-R (0.659, 95% CI 0.565–0.748), but the difference was small and not statistically significant (difference −0.039, *p* = 0.44). Thus, in SMF the disease-centered DIPSS-R recovers most of the prognostic information captured by the dedicated MYSEC-PM.

Reclassification between the DIPSS-R and the MYSEC-PM was also substantial (Supplementary Figure S3C; Supplementary Table S6). The DIPSS-R classified more SMF patients as higher-risk than the MYSEC-PM (62.2% vs. 50.0%; McNemar *p* = 0.07). The two systems agreed for 55 patients (74.3%; 23 concordantly lower-risk and 32 concordantly higher-risk) and disagreed for 19 (25.7%). Both discordant groups had worse survival than concordantly lower-risk patients (5-year OS 85.9%), although to differing degrees (Supplementary Figure S3D). The 14 patients up-stratified by the DIPSS-R relative to the MYSEC-PM had an intermediate course (5-year OS 62.9%; median 106.3 months) that did not reach statistical significance relative to concordantly lower-risk patients (HR 2.52, 95% CI 0.79–8.10; log-rank *p* = 0.11). In contrast, the five patients up-stratified by the MYSEC-PM (and considered lower-risk by the DIPSS-R) had significantly worse survival (5-year OS 30.0%; median 51.1 months; HR 5.30, 95% CI 1.06–26.52; log-rank *p* = 0.02), comparable to concordantly higher-risk patients. Thus, in SMF the MYSEC-PM recognizes a small subset of higher-risk patients that the DIPSS-R under-stages, consistent with its marginally higher C-index, although the numbers are small and these observations are hypothesis-generating.

### 3.7. Independent Prognostic Value and Host Factors

In a multivariable Cox model adjusting for age, the Charlson comorbidity index, MF subtype, sex and JAK2 status (Supplementary Table S7), the DIPSS-R remained strongly and independently prognostic (HR 1.96 per risk category, 95% CI 1.58–2.44; *p* < 0.001). Independently of the DIPSS-R, the Charlson comorbidity index also retained prognostic value (HR 1.18 per point, 95% CI 1.06–1.32; *p* = 0.003), whereas age > 65 years did not reach independent significance (HR 1.49, 0.98–2.28; *p* = 0.064). Thus, although the DIPSS-R captures disease-related risk independently of age, age and comorbidity continue to carry prognostic information, supporting the use of the DIPSS-R as a disease-centered complement to, rather than a replacement of, host-based assessment.

### 3.8. Sensitivity and Landmark Analyses

Restricting the analysis to PMF, for which both scores were originally developed, preserved comparable discrimination (C-index DIPSS 0.731 vs. DIPSS-R 0.712), and both models discriminated less well in SMF (0.622 vs. 0.644), where the DIPSS-R was numerically superior, particularly in post-PV SMF (0.695 vs. 0.643). Landmark analyses confirmed that prognostic information was retained dynamically: among 12-month survivors the C-indices were 0.689 (DIPSS) and 0.687 (DIPSS-R), and among 24-month survivors 0.704 and 0.718, respectively. Survival curves by myelofibrosis subtype are provided in Supplementary Figure S4. Together, these analyses indicate that the comparable performance of the two systems is robust across disease subtypes and over the disease course.

## 4. Discussion

In this multinational, multicenter cohort of 285 patients with MF, the newly proposed age-agnostic DIPSS-R matched the established DIPSS in overall discriminatory accuracy and calibration, with no significant difference in C-index, time-dependent AUC or IDI either in the overall cohort or in any clinically relevant subgroup. The principal contribution of the DIPSS-R lay not in superior discrimination but in reclassification: it assigned markedly more patients to higher-risk categories. The patients it up-stratified, all of whom the DIPSS had classified as lower-risk, experienced a significantly worse clinical course. This effect was most pronounced among transplant-eligible patients aged ≤65 years, more than one-fifth of whom were reclassified upward.

By design, the DIPSS-R replaces the host-related age term and the subjective constitutional-symptoms term with additional disease-intrinsic variables (monocytosis, thrombocytopenia and a lower leukocytosis threshold). Our analysis of discordant patients demonstrates that these same variables drove the upward reclassification: patients up-stratified by the DIPSS-R exhibited higher monocyte and leukocyte counts, lower platelet counts and more frequent transfusion dependence than concordantly lower-risk patients. Monocytosis and thrombocytopenia are recognized markers of aggressive, often more proliferative or fibrotic, disease [26] and are not captured by the DIPSS, which plausibly accounts for the under-recognition of risk in a subset of patients, particularly younger patients, in whom the age point cannot accrue [12,16]. The biological rationale for incorporating the absolute monocyte count into the DIPSS-R is well grounded. Monocytosis in myelofibrosis reflects a granulomonocytic skewing of clonal hematopoiesis and an inflammatory cytokine milieu, frequently accompanies a more proliferative phenotype [27], and may herald features overlapping with chronic myelomonocytic leukemia. Monocytosis was shown to be an independent adverse prognostic factor for survival that was particularly pronounced in younger patients with primary myelofibrosis [26].

Our findings both validate and contextualize the original DIPSS-R report [16]. We confirm that the DIPSS-R is a valid prognostic tool in an independent, real-world, multinational cohort that includes pre-fibrotic PMF and SMF (populations under-represented in score-development cohorts). At the same time, the overall discriminatory accuracy we observed was somewhat lower than in the developmental cohort (C-index approximately 0.69 vs. 0.78), as is typical of external validation, and we found no discrimination advantage for the DIPSS-R over the DIPSS. This reframes the contribution of the DIPSS-R from that of a more accurate score to that of a complementary, disease-centered instrument that reclassifies clinically important patients. The clinical implications are concentrated in the transplant-eligible population. Decisions regarding alloSCT depend on identifying patients whose disease-related risk justifies the morbidity and mortality of the procedure, ideally before irreversible disease progression [7]. In younger patients, the dominant weight of age in the DIPSS is, by definition, absent, so disease-related risk must be inferred from a comparatively small number of variables and by additional use of genetics-based tools. Our data suggest that the DIPSS may consequently leave a clinically important fraction of younger patients under-staged, and that the DIPSS-R recovers this information.

The subgroup reclassification analysis further demonstrates that the additional up-stratification produced by the DIPSS in older patients did not track with worse survival, reinforcing the rationale for an age-agnostic, disease-centered model. A sex difference was also observed (significant reclassification in men but not in women), which, although based on small numbers and uncorrected for multiple comparisons, warrants confirmation.

In secondary myelofibrosis, the DIPSS-R discriminated comparably to the disease-specific MYSEC-PM, with no statistically significant difference between the two (C-index 0.659 vs. 0.698; *p* = 0.44), indicating that a disease-centered design generalizes reasonably from primary to secondary disease. Nonetheless, the small subset of patients up-stratified by the MYSEC-PM but not by the DIPSS-R experienced a poorer outcome, suggesting that the dedicated model may still capture a limited amount of secondary-disease-specific risk. The MYSEC-PM was derived specifically in post-PV/post-ET myelofibrosis and weights variables calibrated to secondary disease, including CALR mutational status, age, hemoglobin, platelet count and circulating blasts. The five patients up-stratified by the MYSEC-PM but considered lower-risk by the DIPSS-R may therefore carry secondary-disease-specific adverse features [28] that the DIPSS-R, lacking a molecular term and not tailored to secondary disease, does not weight. Because this concerns only five patients, the finding is hypothesis-generating and indicates that the MYSEC-PM may retain incremental value in secondary myelofibrosis, where a disease-specific model should still be preferred [29].

Finally, multivariable analysis confirmed the DIPSS-R to be independently prognostic to the Charlson comorbidity index. This argues for pairing the disease-centered DIPSS-R with an explicit assessment of host fitness (for example, a comorbidity index) [30] when making intensive-treatment decisions, rather than relying on either dimension alone.

Our study has several limitations. Its retrospective, multicenter design carries inherent potential for selection bias and treatment-related confounding, as therapy administered over a long, heterogeneous follow-up was not standardized and could not be incorporated into the survival models; lower-risk patients are typically managed more conservatively and higher-risk patients more intensively, which may influence outcomes independently of baseline risk. Comprehensive molecular profiling was unavailable for most patients, several subgroups were small (e.g., the SMF cohort), limiting statistical power, and the entirely Caucasian cohort constrains wider generalizability. Because numerous subgroup and reclassification comparisons were performed without formal correction for multiple testing, some associations—most notably the sex-related difference in reclassification—should be regarded as hypothesis-generating. Transplant outcomes and treatment decisions were not directly evaluated, so the clinical implications of DIPSS-R-based reclassification, particularly for the timing of allogeneic transplantation, remain to be confirmed prospectively. Finally, while the cohort provides an independent external validation of the DIPSS-R, our reclassification findings (including the concordant and discordant analyses) were not confirmed in a separate series and warrant validation in additional cohorts.

## 5. Conclusions

The age-agnostic DIPSS-R performed as well as the DIPSS in discriminating overall survival across all examined subgroups, while reclassifying a substantial proportion of patients into higher-risk categories. The patients up-stratified by the DIPSS-R had significantly worse survival than patients both systems considered lower-risk, driven by disease-related features (monocytosis, leukocytosis, thrombocytopenia) that the DIPSS does not capture. As a disease-centered complement to the DIPSS, the DIPSS-R may improve the recognition of adverse-risk disease in younger patients, in whom earlier and more intensive therapy, including alloSCT, may be most likely to alter outcomes. In secondary myelofibrosis, the DIPSS-R discriminated comparably to the disease-specific MYSEC-PM, supporting its applicability across both primary and secondary disease. Prospective studies integrating molecular data and transplant endpoints are warranted to confirm the clinical utility of DIPSS-R-based reclassification.

## Figures and Tables

**Table 1:** Baseline characteristics of the overall cohort and stratified by age (≤65 vs. >65 years). Continuous variables are medians (IQR); categorical variables are n (%). The Mann–Whitney U and χ^2^ tests were used.

Characteristic	Overall (n = 285)	Age ≤ 65 (n = 119)	Age > 65 (n = 166)	*p*
Age, years	68.0 (60.0–75.0)	58.0 (52.0–62.0)	73.0 (69.0–77.0)	<0.001
Female sex, n (%)	110 (38.6)	40 (33.6)	70 (42.2)	0.180
pre-PMF, n (%)	90 (31.6)	43 (36.1)	47 (28.3)	0.204
overt PMF, n (%)	106 (37.2)	38 (31.9)	68 (41.0)	0.152
post-PV SMF, n (%)	46 (16.1)	19 (16.0)	27 (16.3)	1.000
post-ET SMF, n (%)	43 (15.1)	19 (16.0)	24 (14.5)	0.855
JAK2 V617F mutated, n (%)	198 (69.5)	75 (63.0)	123 (74.1)	0.061
BM fibrosis grade 2–3, n (%)	195 (68.4)	76 (63.9)	119 (71.7)	0.204
Hemoglobin, g/L	114.0 (94.0–137.0)	122.0 (96.0–143.0)	107.5 (92.0–129.8)	0.014
WBC, ×10^9^/L	10.9 (6.8–17.6)	10.3 (6.0–16.4)	11.4 (7.6–18.1)	0.072
ANC, ×10^9^/L	7.4 (3.9–12.6)	6.8 (3.4–12.1)	8.1 (4.5–14.3)	0.023
ALC, ×10^9^/L	1.5 (1.1–2.2)	1.5 (1.1–2.3)	1.5 (1.1–2.2)	0.716
AMC, ×10^9^/L	0.5 (0.3–0.9)	0.4 (0.3–0.7)	0.6 (0.3–1.0)	0.012
Platelets, ×10^9^/L	366.0 (204.0–603.0)	336.0 (221.5–574.0)	395.0 (199.0–672.5)	0.212
PB blasts, %	0.0 (0.0–0.0)	0.0 (0.0–0.0)	0.0 (0.0–0.0)	0.775
LDH, U/L	423.0 (297.8–647.0)	389.0 (261.0–624.0)	452.0 (323.0–650.0)	0.126
CRP, mg/L	4.5 (1.3–11.8)	4.4 (1.4–10.6)	4.7 (1.3–12.4)	0.942
Charlson comorbidity index	3.0 (2.0–4.0)	2.0 (1.0–3.0)	4.0 (3.0–5.0)	<0.001
Transfusion-dependent, n (%)	68 (23.9)	21 (17.6)	47 (28.3)	0.052
Constitutional symptoms, n (%)	129 (45.3)	49 (41.2)	80 (48.2)	0.292

Abbreviations: PMF, primary myelofibrosis; SMF, secondary myelofibrosis; PV, polycythemia vera; ET, essential thrombocythemia; BM, bone marrow; WBC, white blood cells; ANC/ALC/AMC, absolute neutrophil/lymphocyte/monocyte count; LDH, lactate dehydrogenase; CRP, C-reactive protein; PB, peripheral blood; IQR, interquartile range.

**Table 2:** Characteristics of patients by concordance/discordance between the DIPSS and the DIPSS-R. Continuous variables are medians (IQR); categorical variables are n (%).

Characteristic	Concordant Lower (n = 99)	DIPSS-R Up-Strat. (n = 46)	DIPSS Up-Strat. (n = 11)	Concordant Higher (n = 114)	*p*
Age, years	61.0 (53.0–70.5)	64.0 (58.0–70.8)	76.0 (70.5–78.5)	69.5 (65.0–77.0)	<0.001
Female sex, n (%)	41 (41.4)	12 (26.1)	3 (27.3)	48 (42.1)	0.207
Secondary MF, n (%)	31 (31.3)	15 (32.6)	2 (18.2)	37 (32.5)	0.806
JAK2 V617F mutated, n (%)	65 (65.7)	37 (80.4)	8 (72.7)	78 (68.4)	0.333
BM fibrosis grade 2–3, n (%)	46 (46.5)	29 (63.0)	7 (63.6)	100 (87.7)	<0.001
Hemoglobin, g/L	134.0 (121.0–148.0)	120.0 (110.0–137.8)	97.0 (96.0–115.0)	91.0 (83.0–99.0)	<0.001
WBC, ×10^9^/L	9.6 (6.9–11.8)	16.1 (12.4–25.7)	8.3 (6.5–9.4)	11.3 (5.8–25.7)	<0.001
ANC, ×10^9^/L	6.5 (4.3–9.4)	12.3 (8.1–21.3)	4.8 (4.3–6.5)	7.3 (2.9–15.0)	<0.001
AMC, ×10^9^/L	0.5 (0.3–0.6)	0.9 (0.4–1.2)	0.4 (0.3–0.6)	0.5 (0.2–1.1)	0.002
Platelets, ×10^9^/L	528.0 (311.0–722.0)	387.0 (150.0–682.2)	387.0 (302.5–527.0)	234.0 (114.8–426.2)	<0.001
PB blasts, %	0.0 (0.0–0.0)	0.0 (0.0–0.0)	0.0 (0.0–0.0)	0.0 (0.0–0.0)	<0.001
LDH, U/L	317.0 (229.0–481.0)	463.5 (293.2–663.0)	352.0 (238.0–560.5)	498.5 (364.2–799.2)	<0.001
Charlson comorbidity index	2.0 (2.0–4.0)	2.0 (2.0–4.0)	5.0 (3.0–5.0)	4.0 (3.0–5.0)	<0.001
Transfusion-dependent, n (%)	0 (0.0)	5 (10.9)	0 (0.0)	61 (53.5)	<0.001
Constitutional symptoms, n (%)	17 (17.2)	16 (34.8)	8 (72.7)	83 (72.8)	<0.001
5-year OS, %	81.7	57.7	54.5	23.7	—
Median OS, months	128.5	84.1	75.4	38.4	—

Across-group comparison by Kruskal–Wallis (continuous) or χ^2^ (categorical) test. Abbreviations: PMF, primary myelofibrosis; SMF, secondary myelofibrosis; PV, polycythemia vera; ET, essential thrombocythemia; BM, bone marrow; WBC, white blood cells; ANC/ALC/AMC, absolute neutrophil/lymphocyte/monocyte count; LDH, lactate dehydrogenase; CRP, C-reactive protein; PB, peripheral blood; IQR, interquartile range; up-strat., up-stratified.

## Data Availability

The data supporting the findings of this study are available from the corresponding author upon reasonable request.

## Supplementary Materials

The following supporting information can be downloaded at: https://www.mdpi.com/article/10.3390/cancers18132159/s1, Figure S1: Time-dependent discrimination and calibration of the DIPSS and the DIPSS-R; Figure S2: Overall survival by risk category in patients aged ≤65 years; Figure S3: Overall survival and risk reclassification in secondary myelofibrosis; Figure S4: Overall survival by risk category in overt and pre-fibrotic PMF; Table S1: Discriminatory accuracy of the DIPSS and the DIPSS-R across subgroups; Table S2: Risk-category distribution and cross-system reclassification; Table S3: Cross-classification of the DIPSS and the DIPSS-R; Table S4: Subgroup reclassification analysis; Table S5: Comparison of the DIPSS-R and the MYSEC-PM in secondary MF; Table S6: Cross-classification of the MYSEC-PM and the DIPSS-R in secondary MF; and Table S7: Multivariable Cox regression for overall survival.
