# Supplementary material for: Comparative Value of the Novel Age-Agnostic DIPSS-R Versus the DIPSS for Prognostication in Myelofibrosis: A Multicenter Evaluation and Reclassification Study
Source: Cancers (Basel). 2026 Jul 5;18(13):2159. doi: 10.3390/cancers18132159 (PMC13359450; doi:10.3390/cancers18132159)
Supplement: Supplementary file 1 [file cancers-18-02159-s001.zip › cancers-4379867-supplementary.pdf]

Supplementary Materials: Comparative Value of the Novel Age-Agnostic DIPSS-R versus the DIPSS for Prognostication in Myelofibrosis: A Multicenter Evaluation and Reclassification Study

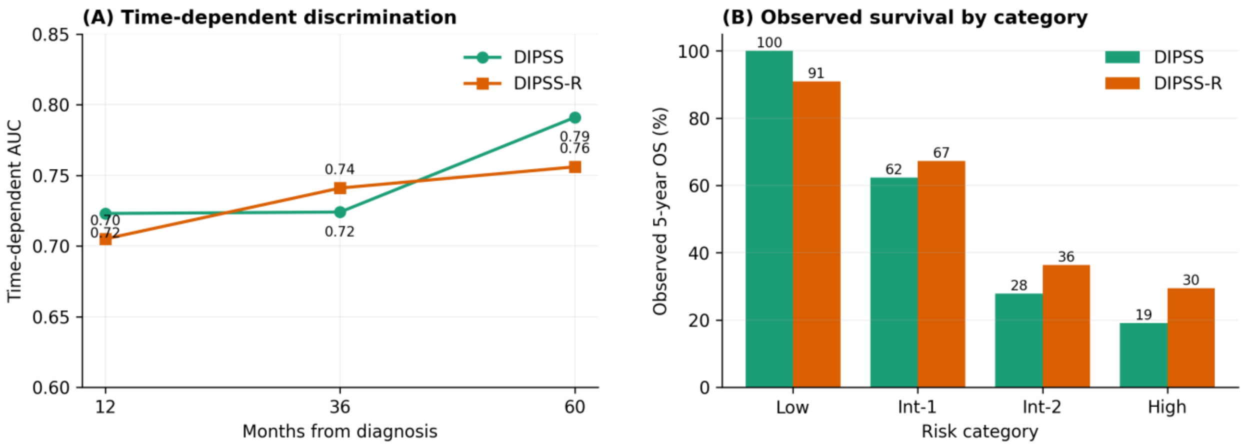

Figure S1. (**A**) Time-dependent area under the ROC curve at 12, 36 and 60 months for the DIPSS and the DIPSS-R. (**B**) Observed (Kaplan-Meier) 5-year overall survival by risk category for each system.

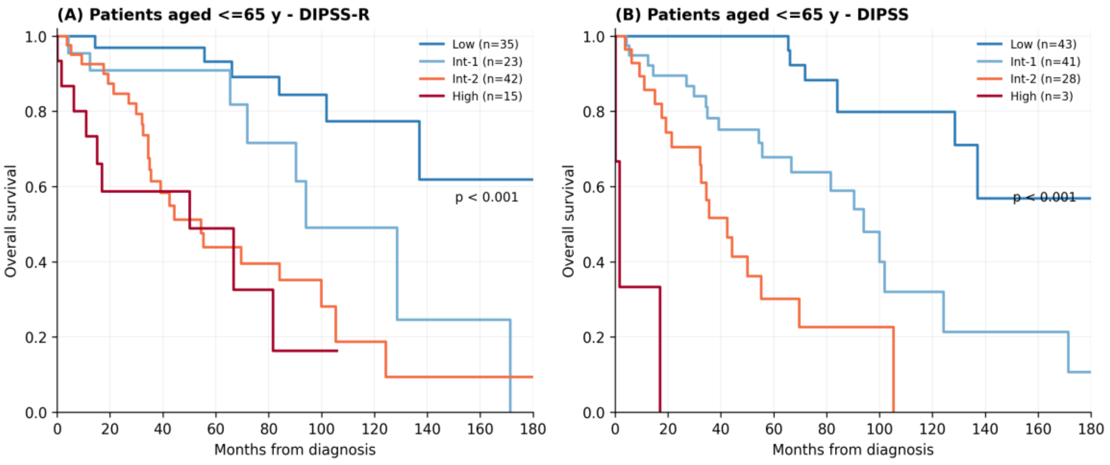

Figure S2. Kaplan-Meier overall survival stratified by the DIPSS-R (**A**) and the DIPSS (**B**) in transplant-eligible patients aged ≤65 years.

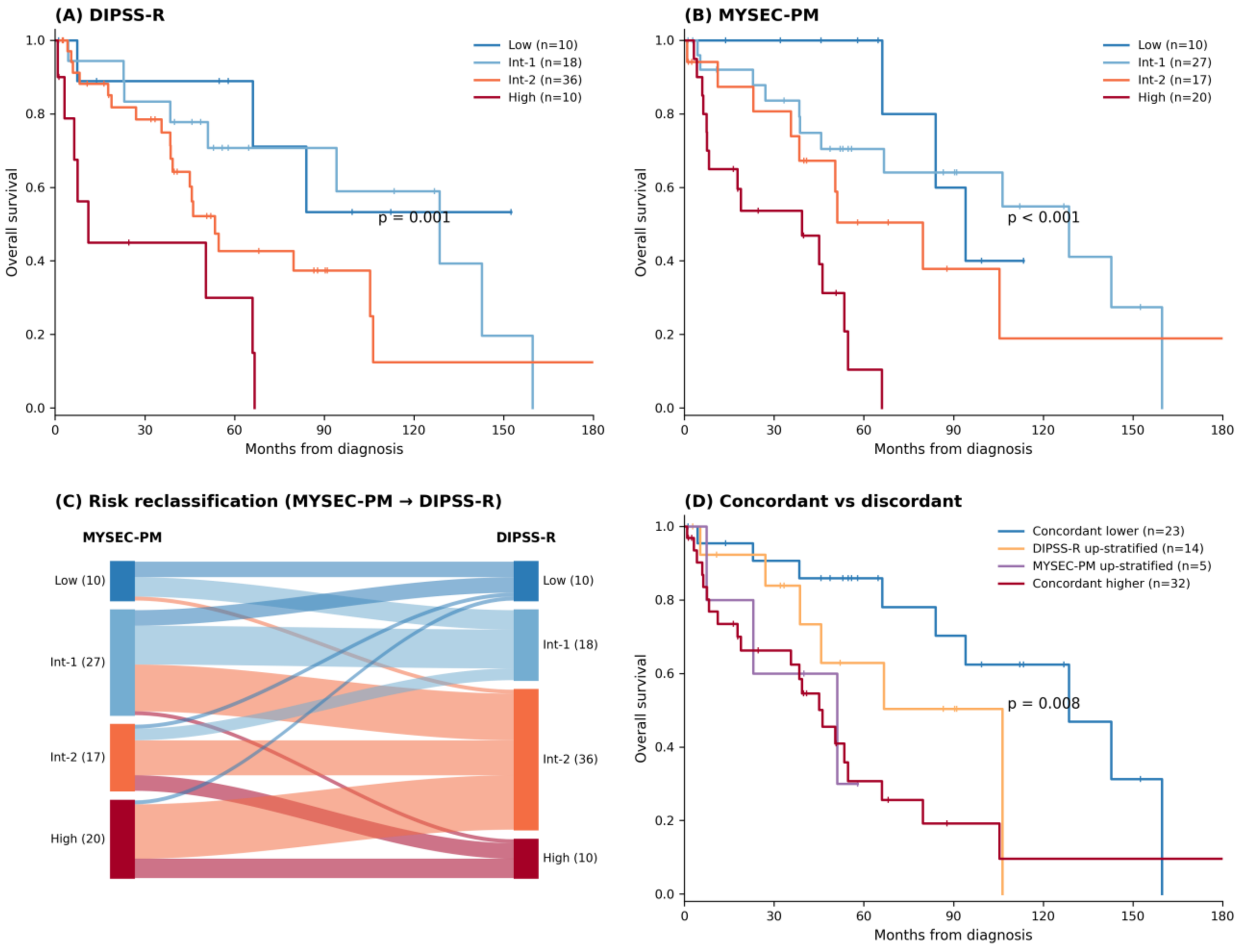

Figure S3. Kaplan-Meier estimates of overall survival and risk reclassification in secondary myelofibrosis (n = 74). Overall survival stratified by (**A**) the DIPSS-R and (**B**) the MYSEC-PM. (**C**) Alluvial diagram of risk reclassification from the MYSEC-PM (left) to the DIPSS-R (right); ribbon colour denotes the DIPSS-R category. (**D**) Overall survival by concordance/discordance between the two systems.

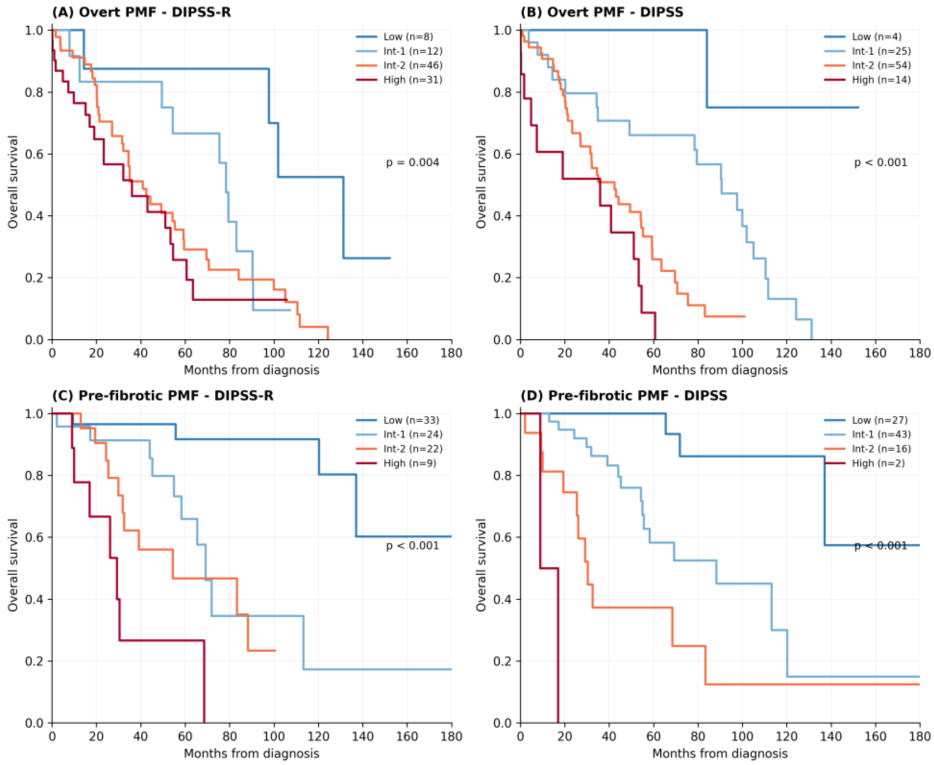

Figure S4. Kaplan-Meier overall survival stratified by the DIPSS-R and the DIPSS in overt PMF (**A**,**B**) and in pre-fibrotic PMF (**C**,**D**).

Table S1. Discriminatory accuracy (Harrell's C-index, 95% CI) of the DIPSS and the DIPSS-R across subgroups, in the comparative analysis set (n = 270). Overlapping 95% CIs indicate no significant difference between systems.

Subgroup	n	Deaths	DIPSS	DIPSS-R
**Overall**	270	148	0.697 (0.655-0.737)	0.691 (0.644-0.739)
PMF (all)	185	103	0.731 (0.686-0.778)	0.712 (0.662-0.765)
pre-PMF	88	32	0.789 (0.718-0.864)	0.772 (0.684-0.856)
overt PMF	97	71	0.644 (0.568-0.712)	0.622 (0.545-0.693)
SMF (all)	85	45	0.622 (0.532-0.716)	0.644 (0.546-0.728)
post-PV SMF	44	23	0.643 (0.524-0.757)	0.695 (0.586-0.801)
post-ET SMF	41	22	0.591 (0.471-0.718)	0.601 (0.450-0.743)
BM fibrosis grade 0-1	88	32	0.789 (0.714-0.852)	0.772 (0.683-0.861)
BM fibrosis grade 2-3	182	116	0.640 (0.581-0.696)	0.640 (0.586-0.693)
Age ≤65	115	48	0.778 (0.715-0.834)	0.736 (0.657-0.807)
Age >65	155	100	0.599 (0.538-0.663)	0.640 (0.574-0.703)
Male	166	91	0.694 (0.638-0.754)	0.695 (0.638-0.753)
Female	104	57	0.703 (0.630-0.765)	0.686 (0.606-0.754)
JAK2 mutated	188	98	0.682 (0.621-0.737)	0.666 (0.602-0.721)
JAK2 wild-type	74	44	0.736 (0.669-0.806)	0.750 (0.677-0.815)

CI, confidence interval; PMF, primary myelofibrosis; pre-PMF, pre-fibrotic primary myelofibrosis; SMF, secondary myelofibrosis; PV, polycythemia vera; ET, essential thrombocythemia; BM, bone marrow.

Table S2. Risk-category distribution and cross-system reclassification (comparative analysis set, n = 270).

Risk category	DIPSS, n (%)	DIPSS-R, n (%)
Low	43 (15.9)	52 (19.3)
Intermediate-1	102 (37.8)	58 (21.5)
Intermediate-2	102 (37.8)	107 (39.6)
High	23 (8.5)	53 (19.6)
**Higher-risk (Int-2 + High)**	**125 (46.3)**	**160 (59.3)**

Table S3. Cross-classification of patients by the DIPSS (rows) and the DIPSS-R (columns), n = 270. For concordance assessment, categories are collapsed into lower-risk (Low, Int-1) and higher-risk (Int-2, High). Shaded top-left and bottom-right blocks indicate concordant classification; the top-right block (bold) denotes patients up-stratified by the DIPSS-R and the bottom-left block patients up-stratified by the DIPSS.

DIPSS ↓ / DIPSS-R →	Low	Int-1	Int-2	High
**Low**	27	13	**3**	**0**
**Int-1**	24	35	**37**	**6**
**Int-2**	1	10	61	30
**High**	0	0	6	17

Table S4. Subgroup reclassification analysis. For each subgroup: numbers up-stratified by the DIPSS-R and by the DIPSS, and the hazard ratio (HR) for death of DIPSS-R up-stratified patients versus concordantly lower-risk patients.

Subgroup	n	DIPSS-R up, n (%)	DIPSS up, n (%)	HR (95% CI)	p
**Overall**	270	46 (17.0)	11 (4.1)	2.50 (1.49-4.17)	**<0.001**
Pre-fibrotic PMF	88	17 (19.3)	4 (4.5)	3.89 (1.43-10.56)	**0.008**
Overt PMF	97	14 (14.4)	5 (5.2)	1.47 (0.60-3.56)	0.397
Post-PV SMF	44	10 (22.7)	1 (2.3)	3.37 (0.80-14.17)	0.098
Post-ET SMF	41	5 (12.2)	1 (2.4)	1.15 (0.28-4.68)	0.847
PMF (all)	185	31 (16.8)	9 (4.9)	2.79 (1.46-5.34)	**0.002**
SMF (all)	85	15 (17.6)	2 (2.4)	1.68 (0.68-4.15)	0.262
BM fibrosis 0-1	88	17 (19.3)	4 (4.5)	3.89 (1.43-10.56)	**0.008**
BM fibrosis 2-3	182	29 (15.9)	7 (3.8)	1.75 (0.94-3.26)	0.076
Age <=65 y	115	26 (22.6)	0 (0.0)	3.13 (1.46-6.72)	**0.003**
Age >65 y	155	20 (12.9)	11 (7.1)	2.05 (0.99-4.23)	0.052
Age <=70 y	170	34 (20.0)	3 (1.8)	2.97 (1.58-5.58)	**0.001**
Age >70 y	100	12 (12.0)	8 (8.0)	1.97 (0.78-4.98)	0.152
Male	166	34 (20.5)	8 (4.8)	3.46 (1.75-6.85)	**<0.001**
Female	104	12 (11.5)	3 (2.9)	1.41 (0.57-3.45)	0.456
JAK2 mutated	188	37 (19.7)	8 (4.3)	2.67 (1.44-4.95)	**0.002**
CALR mutated	23	2 (8.7)	1 (4.3)	not estimable	-
Non-JAK2/CALR	43	4 (9.3)	2 (4.7)	1.51 (0.29-7.94)	0.625
Transfusion-independent	204	41 (20.1)	11 (5.4)	2.52 (1.48-4.28)	**0.001**
Transfusion-dependent	66	5 (7.6)	0 (0.0)	not estimable	-

Up-stratified = classified higher-risk (Int-2/High) by one system but lower-risk (Low/Int-1) by the other. HR estimated by Cox regression vs. concordantly lower-risk patients. Transfusion-dependent patients cannot be DIPSS-R lower-risk and are therefore omitted.

Table S5. Comparison of the DIPSS-R and the MYSEC-PM in secondary MF (n = 74, 40 deaths).

Score	C-index (95% CI)	Pairwise comparison
MYSEC-PM	0.698 (0.620-0.773)	-
DIPSS-R	0.659 (0.565-0.748)	DIPSS-R vs. MYSEC-PM: difference -0.039, p = 0.44

Table S6. Cross-classification of secondary-MF patients by the MYSEC-PM (rows) and the DIPSS-R (columns), n = 74. For concordance, categories are collapsed into lower-risk (Low, Int-1) and higher-risk (Int-2, High). Shaded top-left and bottom-right blocks = concordant; top-right block (bold) = up-stratified by the DIPSS-R relative to the MYSEC-PM.

MYSEC-PM ↓ / DIPSS-R →	Low	Int-1	Int-2	High
**Low**	4	5	**1**	**0**
**Int-1**	4	10	**12**	**1**
**Int-2**	1	3	9	4
**High**	1	0	14	5

Table S7. Multivariable Cox regression for overall survival including the DIPSS-R and host/disease covariates (n = 201, 118 deaths; model C-index 0.707).

Variable	HR (95% CI)	p
DIPSS-R (per risk category)	1.96 (1.58-2.44)	**<0.001**
Age > 65 years	1.49 (0.98-2.28)	0.064
Charlson comorbidity index (per point)	1.18 (1.06-1.32)	**0.003**
Secondary MF (vs. PMF)	0.94 (0.63-1.41)	0.773
Male sex	1.14 (0.77-1.68)	0.513
JAK2 V617F mutation	0.87 (0.59-1.28)	0.472

HR, hazard ratio. In a model replacing the DIPSS-R with the DIPSS, the DIPSS was likewise independent (HR 2.22, 1.74-2.83; p < 0.001) with an equivalent C-index (0.708).
